# The AMPA receptor antagonist perampanel robustly rescues amyotrophic lateral sclerosis (ALS) pathology in sporadic ALS model mice

**DOI:** 10.1038/srep28649

**Published:** 2016-06-28

**Authors:** Megumi Akamatsu, Takenari Yamashita, Naoki Hirose, Sayaka Teramoto, Shin Kwak

**Affiliations:** 1 Center for Disease Biology and Integrative Medicine, Graduate School of Medicine, University of Tokyo, Bunkyo-ku, Tokyo, Japan; 2 Department of Neuropathology, Graduate School of Medicine, University of Tokyo, Bunkyo-ku, Tokyo, Japan; 3 Clinical Research Center for Medicine, International University of Health and Welfare, Ichikawa, Chiba, Japan

## Abstract

Both TDP-43 pathology and failure of RNA editing of AMPA receptor subunit GluA2, are etiology-linked molecular abnormalities that concomitantly occur in the motor neurons of the majority of patients with amyotrophic lateral sclerosis (ALS). AR2 mice, in which an RNA editing enzyme adenosine deaminase acting on RNA 2 (ADAR2) is conditionally knocked out in the motor neurons, exhibit a progressive ALS phenotype with TDP-43 pathology in the motor neurons through a Ca^2+^-permeable AMPA receptor-mediated mechanism. Therefore, amelioration of the increased Ca^2+^ influx by AMPA receptor antagonists may be a potential ALS therapy. Here, we showed that orally administered perampanel, a selective, non-competitive AMPA receptor antagonist significantly prevented the progression of the ALS phenotype and normalized the TDP-43 pathology-associated death of motor neurons in the AR2 mice. Given that perampanel is an approved anti-epileptic drug, perampanel is a potential candidate ALS drug worthy of a clinical trial.

Amyotrophic lateral sclerosis (ALS) is the most common adult-onset motor neuron disease, characterized by the progressive loss of both upper and lower motor neurons. Patients with ALS die from progressive respiratory muscle paralysis within a few years after disease onset, but therapies that effectively alter the disease course are not currently available. Although more than 30 ALS-linked genes, including the Cu/Zn superoxide dismutase (*SOD1*) gene, have been identified^1^, most of the sporadic ALS patients that account for the great majority of all the ALS patients do not carry mutations in these ALS-linked genes. The candidate drugs that have been developed to treat ALS are largely based on studies in SOD1 transgenic mice^2^,^3^,^4^, the most widely used ALS animal model, but virtually all of these drugs have proven ineffective^5^,^6^,^7^. Therefore, a novel strategy for drug development that exploits evaluation markers that are closely linked with ALS pathogenesis would be valuable.

TAR DNA-binding protein 43 (TDP-43) pathology, which is characterized by the mislocalization of TDP-43 from the nucleus to abnormal cytoplasmic inclusion bodies in motor neurons^8^,^9^, is observed in the motor neurons of the vast majority of ALS patients and hence is a pathological hallmark of ALS. The mechanism whereby generating TDP-43 pathology, although has not been fully elucidated, is believed to be closely involved in the ALS pathogenesis. Normalization of TDP-43 pathology could therefore be a reliable marker for therapeutic efficacy, but the motor neurons of SOD1 transgenic mice do not exhibit typical TDP-43 pathology^3^,^10^.

In addition to TDP-43 pathology^8^,^9^, RNA editing failure at the glutamine/arginine (Q/R) site of GluA2, an α-amino-3-hydroxy-5-methyl-4-isoxazole propionic acid (AMPA) receptor subunit, is observed in the motor neurons of the majority of patients with sporadic ALS in a disease-specific manner^11^,^12^,^13^,^14^. Adenosine deaminase acting on RNA 2 (ADAR2) specifically catalyzes RNA editing at the Q/R site of GluA2^15^,^16^,^17^ and is downregulated in the ALS motor neurons^18^. Consistently, in ALS patients, motor neurons that exhibited cytoplasmic inclusions positive for phosphorylated TDP-43 or other patterns of TDP-43 pathology were invariably devoid of ADAR2 immunoreactivity^18^. Conditional ADAR2 knockout mice (ADAR2^flox/flox^/VAChT-Cre.Fast; AR2) exhibit a progressive ALS phenotype^19^,^20^ resulting from the death of motor neurons due to ADAR2-downregulation, and notably exhibit mislocalization of TDP-43 from the nucleus to the cytoplasm in the ADAR2-lacking motor neurons^21^. These lines of evidence indicate that a Ca^2+^-permeable AMPA receptor-mediated mechanism similar to the mechanism that takes place in AR2 mice likely also occurs in the motor neurons of ALS patients^22^. Furthermore, normalization of Ca^2+^ influx through the AMPA receptors, including expression of Q/R site-edited GluA2 in the absence of ADAR2 or delivery of the human ADAR2 gene for normalizing RNA editing at the GluA2 Q/R site^19^,^21^, effectively rescued the ALS phenotype and death of motor neurons associated with the TDP-43 mislocalization in the AR2 mice^19^,^20^. Therefore, the exaggerated Ca^2+^ influx through the abnormal AMPA receptors could be a therapeutic target for sporadic ALS, and the normalization of TDP-43 mislocalization in AR2 mice could serve as a reliable marker to evaluate the efficacy of newly developed treatments^22^.

## Results

### Perampanel prevented the death of motor neurons and normalized TDP-43 subcellular localization in AR2 and AR2H mice

**Figure 1:**
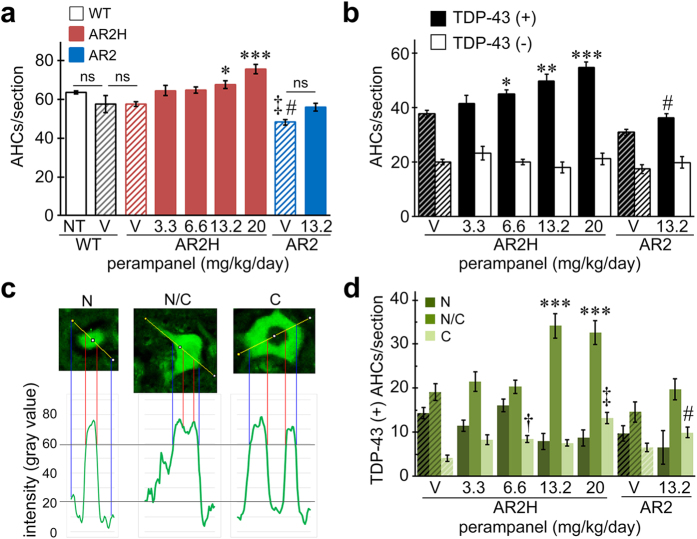
Perampanel administration for 14 days prevented the death of motor neurons and normalized TDP-43 subcellular localization in AR2H (heterozygous) and AR2 (homozygous) mice. (**a**) Number of TO-PRO-3-positive anterior horn cells in the spinal cord (AHCs; ≥20 μm in diameter). Data for the WT, AR2H and AR2 groups are indicated in different colors (white, red, and blue, respectively), and the vehicle groups (V; methyl cellulose) are represented as hatched columns in the corresponding colors. NT, no treatment. *p < 0.05 and ***p < 0.0001 against the AR2H-V group; ^#^*p* < 0.05 against the AR2H-V group; and ^‡^*p* < 0.0001 against the WT group. ns, not statistically significant. (**b**) Number of TDP-43-positive (black columns) and TDP-43-negative (white columns) AHCs. Hatched columns indicate the results on vehicle-treated mice. *p < 0.05, **p < 0.001, and ***p < 0.0001 against the AR2H-V group; and ^#^*p* < 0.05 against the AR2-V group. (**c**) Representative AHCs with different TDP-43 subcellular localization patterns: predominantly nuclear (N), nucleo-cytoplasmic (N/C) or cytoplasmic (C). The vertical axis indicates the intensity (gray value) evaluated with ImageJ. The threshold for TDP-43 positivity was set at a level 3-fold higher (60 gray) than the background intensity (20 gray). (**d**) The numbers of AHCs with different subcellular TDP-43 localization patterns are indicated. Hatched columns indicate the results on vehicle-treated mice. ***p < 0.0001 against the AR2H-V with N/C group; ^†^*p* < 0.05 and ^‡^*p* < 0.0001 against the AR2H-V with C group; and ^#^*p* < 0.05 against the AR2-V with C group, Wilcoxon rank sum test. (**a,b,d**) Data are presented as the mean ± s.e.m. Statistics were based on one-way ANOVA and Tukey *post hoc* tests unless indicated otherwise. In the WT group, 5 (NT) and 3 (V) mice were included; in the AR2H group, 7 (V), 5 (3.3 mg/kg/day), 8 (6.6), 5 (13.2) and 6 (20) mice were included; and in the AR2 group, 5 in both the V and 13.2 mg/kg/day groups were included.

We first administered perampanel to homozygous (AR2)^19^ and heterozygous (AR2H)^20^ conditional ADAR2 knockout mice at 17 weeks of age. The perampanel was delivered orally every day for 14 days; methyl cellulose (vehicle) was used as a control. The mice were akinetic for several hours following the administration, but the duration of the sedative phase became increasingly shorter with time, and all of the mice tolerated the 14-day administration of perampanel at dosages below 20 mg/kg/day. At 19 weeks of age, the number of TO-PRO-3-positive anterior horn cells (AHCs; diameter ≥20 μm) in the 5^th^ lumbar spinal cord (L5) was significantly lower in the vehicle-treated AR2 mice (48.2 ± 1.3, mean ± s.e.m.) than in the AR2H mice (57.7 ± 1.3) and the wild-type (WT) mice (63.5 ± 0.8) (Fig. 1a), in accordance with the results observed for the non-treated AR2 mice^19^,^20^,^23^. In the AR2H mice, perampanel significantly increased the total number of AHCs at dosages greater than 13.2 mg/kg/day (Fig. 1a) and of TDP-43-positive AHCs at dosages greater than 6.6 mg/kg/day, and these increases occurred in a dose-dependent manner (Fig. 1b). In the AR2 mice, perampanel at a dosage of 13.2 mg/kg/day significantly increased the number of TDP-43-positive AHCs but not the total number of AHCs (Fig. 1b). When the TDP-43-positive AHCs were classified according to the subcellular distribution of TDP-43 (Fig. 1c), the number of AHCs with nucleocytoplasmic and/or cytoplasmic TDP-43 was significantly increased by the perampanel treatment at dosages greater than 13.2 mg/kg/day (Fig. 1d).

### Amelioration of motor dysfunction by long-term administration of perampanel to AR2 mice

**Figure 2:**
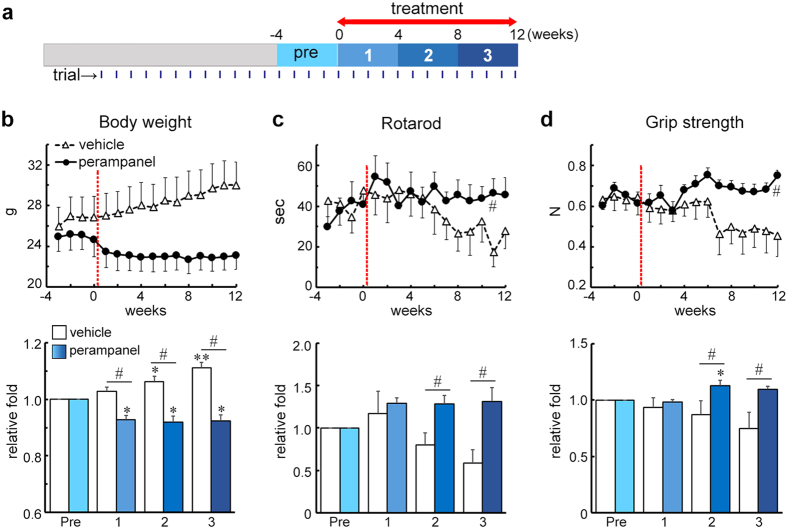
Perampanel administration for 90 days rescued motor dysfunction in AR2 mice. (**a**) The time schedule for performance testing and drug treatment. Body weight (**b**), latency to fall on the rotarod task (**c**) and grip strength (**d**) were examined every week throughout the experiment in the perampanel- (*n* = 8; *n* = 1 of male, *n* = 7 of female) and vehicle- (*n* = 7; *n* = 3 of male, *n* = 4 of female) treated AR2 mice. Weekly performance scores are indicated in the upper panels (red bars indicate the first day of administration). Mean values for the four trials before perampanel treatment (pre), initial four trials (1), from the 5th to the 8th trials (2) and from the 9th to the last trails (3) after perampanel treatment are indicated in the lower panels. All data are presented as the mean ± s.e.m. *p < 0.05, **p < 0.001 against the “pre” time point at each time point, one-way ANOVA and Tukey *post hoc* tests. ^#^*p* < 0.05 perampanel against the vehicle group at each time point, Wilcoxon rank sum test.

To test the efficacy of perampanel on the ALS phenotype, we next orally administered perampanel every day for 90 days to AR2 mice at 26-28 weeks of age, a time when the majority of AR2 mice already exhibit motor dysfunction^19^,^23^ (Fig. 2a). To avoid excessive sedation upon treatment initiation, perampanel was first administered at a dose of 13.2 mg/kg for 4 days and then at 20 mg/kg for the remaining 86 days; methyl cellulose (vehicle) was used as a control. All of the mice were tested for rotarod retention time and grip strength once a week throughout the experiment, beginning several weeks before the first perampanel administration. The perampanel-treated AR2 mice did not gain weight (Fig. 2b) but were behaviorally active and displayed a constant rotarod score (Fig. 2c) and grip strength (Fig. 2d) throughout the experiment, which were significantly better than the vehicle-treated AR2 mice. All of the mice tolerated the 90-day-administration of perampanel.

### Long-term administration of perampanel rescued AHCs from death and normalized TDP-43 subcellular localization

**Figure 3:**
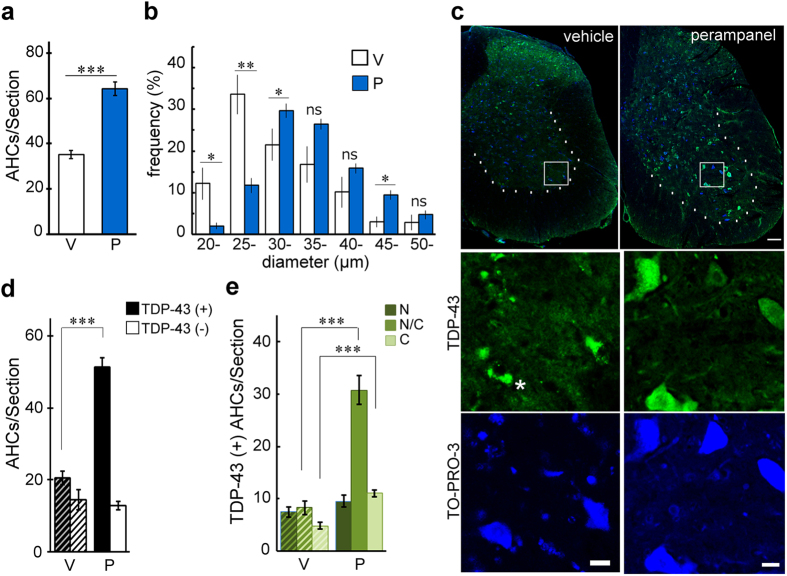
Perampanel administration for 90 days rescued AHCs from death and normalized TDP-43 subcellular localization. (**a**) Number of AHCs in L5. V, vehicle (methyl cellulose)-treated AR2 mice (*n* = 7); P, perampanel-treated AR2 mice (*n* = 8). Three sections were measured for each mouse. (**b**) Frequency histogram of AHCs in the perampanel group (P, blue columns) and the vehicle group (V, white columns). The vertical axis indicates the proportion of the total number of AHCs with a diameter within each range. (**c**) Immunostaining of the lumbar spinal cord for TDP-43 (green). White dotted lines indicate the margin of the ventral gray matter. Asterisk: TDP-43-positive cytoplasmic aggregates. TO-PRO-3 (blue) was used as a cell body marker. The scale bar indicates 100 μm (upper panels) or 20 μm (lower panels). (**d**) Number of TDP-43-positive (black columns) and TDP-43-negative (white columns) AHCs. Hatched columns indicate the results on vehicle-treated mice. ***p < 0.0001 against the vehicle (V) group, Wilcoxon rank sum test. (**e**) The number of AHCs showing TDP-43 immunoreactivity in the nucleus (N), cytoplasm (C), and both the nucleus and cytoplasm (N/C) are indicated. Hatched columns indicate the results on vehicle-treated mice. (**a,b,d,e**) All error bars represent the s.e.m.; *p < 0.05, **p < 0.001, and ***p < 0.0001 against the vehicle (V) group, Wilcoxon rank sum test.

When the spinal cords were histologically evaluated, the number of AHCs in the perampanel-treated AR2 mice (64.2 ± 3.0) was significantly greater than that in the vehicle-treated mice (35.2 ± 1.8, Fig. 3a). TO-PRO-3 was used as a cell marker in our experiments^24^,^25^. In the AR2 mice, perampanel administration also shifted the peak of the histogram of AHC diameter from the range of 25–29 μm to that of 30–34 μm and increased the proportion of AHCs with a diameter greater than 30 μm compared to the vehicle-treated AR2 mice (Fig. 3b). The frequency histogram of AHC diameter revealed that perampanel restored the size of AHCs in the AR2 mice to a level equivalent to that of AHCs in the age-matched WT mice (Supplementary Fig. 1). The number of TDP-43-positive AHCs was also increased in the perampanel-treated AR2 mice (Fig. 3c,d), particularly those that were TDP-43-positive in both the nucleus and cytoplasm (Fig. 3c,e). Furthermore, most of the TDP-43-positive AHCs in the vehicle-treated AR2 mice exhibited focal cytoplasmic TDP-43-positive aggregates or inclusions (asterisk in Fig. 3c) rather than the diffuse cytoplasmic positivity observed in the TDP-43-positive AHCs in the perampanel-treated AR2 mice (Fig. 3c).

## Discussion

Present results showed that oral administration of perampanel, a selective, non-competitive AMPA receptor antagonist^26^,^27^, to the AR2H and AR2 mice for 14 days effectively normalized TDP-43 pathology in motor neurons and that its administration for 90 days significantly prevented the progression of the ALS phenotype and the TDP-43 pathology-associated death of motor neurons. Success in the rescue of ALS phenotype of the AR2 mice, a mechanistic mouse model of sporadic ALS, indicates that perampanel is a potential candidate ALS drug.

Perampanel is a novel AMPA receptor antagonist that inhibits the excitation of postsynaptic membranes by selectively attenuating calcium influx through AMPA receptors^26^. As expected, perampanel increased the number of TDP-43-positive AHCs in the AR2 mice (Figs 1b and 3d). TDP-43 immunoreactivity was observed virtually exclusively in the nucleus in the normal motor neurons of WT mice, whereas it mislocalized in the cytoplasm or was absent in the motor neurons that expressed Q/R site-unedited GluA2 in the AR2 mice^21^. This mislocalization of TDP-43 was caused by abnormal and continuous activation of Ca^2+^-dependent protease calpain and normalization of Ca^2+^ influx through the AMPA receptors restored TDP-43 mislocalization in the AR2 mice^21^. In the present study, we found three different patterns of subcellular localization of TDP-43 in the AR2 mouse motor neurons; nuclear, nucleocytoplasmic, and cytoplasmic. A previous study has shown that these different subcellular distribution patterns of TDP-43 immunoreactivity likely reflected different levels of cytoplasmic Ca^2+^ concentrations in the motor neurons; higher in the cytoplasmic pattern than in the nucleocytoplasmic pattern, and normal in the nuclear pattern^21^. The present results demonstrating the increase in the number of TDP-43-positive neurons, particularly those with nuclear and nucleocytoplasmic patterns (Figs 1d and 3e) suggest that perampanel may have ameliorated the increased Ca^2+^ influx that occurred in the ADAR2-lacking motor neurons in the AR2 mice.

In addition, perampanel, and not vehicle, restored the size of AHCs to the level observed in age-matched WT mice (Fig. 3b); this effect likely reflects the restoration of physiological functions that were disturbed by the increased Ca^2+^ influx through the abnormal AMPA receptors. Notably, perampanel was effective not only in asymptomatic AR2 mice but also in AR2 mice during the progression of the disease, as observed in the study of adeno-associated virus-mediated delivery of the ADAR2 gene^23^. The robust protective effects of perampanel on ADAR2-lacking motor neurons in AR2 mice suggest that perampanel would be even more effective in preventing excess Ca^2+^ influx in the motor neurons of ALS patients because ALS AHCs maintain ADAR2 activity to some extent^14^. The perampanel-treated AR2 mice failed to gain body weight but were physically active and exhibited better motor function scores than the vehicle-treated AR2 mice (Fig. 2). Because the loss of body weight has not been reported as an adverse effects of perampanel in clinical trials^28^, the lack of body weight gain in the perampanel-treated AR2 mice might have resulted from the preservation of physical activity rather than any adverse effects of perampanel.

Excitotoxicity has been proposed to underlie ALS pathogenesis, and AMPA receptor antagonists are believed to have potential as a treatment for ALS. Indeed, the AMPA receptor antagonist talampanel^29^ was initially found to be beneficial for ALS patients in a phase II clinical trial^5^,^30^, but unfortunately, the phase III clinical trial was not extended due to insufficient efficacy^31^. Both talampanel and perampanel are non-competitive AMPA receptor antagonist, but compared to talampanel, perampanel has a much longer terminal half-life (*t*_1/2_) in humans (approximately 105 h^27^) than talampanel (approximately 3–4 hours^29^). Furthermore, perampanel has been approved in over 40 countries as an adjunctive therapy for the treatment of partial seizures with or without secondary generalization^28^. The long *t*_1/2_ and safety of perampanel render it a promising ALS drug.

Recently, failure of GluA2 Q/R site-editing has been demonstrated in the motor neurons of an ALS patient with *FUS*^*P525L*^ mutation^14^,^32^ and in the pathological tissues of ALS patients carrying the *C9ORF72* gene with hexanucleotide repeat expansion^33^, suggesting that efficacy of perampanel would be expected in some form of familial ALS patients, as well. We believe that the present results provide a rationale for clinical trials of perampanel in ALS patients.

## Methods

### Animals

Homozygous (*ADAR2*^*flox/flox*^/VAChT-Cre.Fast; AR2) and heterozygous (*ADAR2*^*flox/*+^/VAChT-Cre.Fast; AR2H) conditional *ADAR2* knockout mice were used in this study^19^,^20^. In these mice, Cre is selectively expressed in motor neurons under the control of the vesicular acetylcholine transporter promoter^34^, ablating the ADAR2^flox^ gene in approximately 50% of motor neurons by the age of five postnatal weeks. As a result, 100% and no more than 30% of GluA2 is unedited at the Q/R site in the motor neurons of AR2 and AR2H mice, respectively. In these mice, the expression of GluA2 that is unedited at the Q/R site results in the slowly progressive death of motor neurons via Ca^2+^-permeable AMPA receptor-mediated mechanisms^19^,^20^. Both genders of AR2 and AR2H mice were used in this study. C57BL/6J mice (Oriental Yeast Co., Ltd.) of the same age were used as the WT control of the same strain. The mice were housed at two to three per cage on a 12 h: 12 h light-dark cycle with free access to food and water. All of the studies were approved by the Committee on Animal Handling at the University of Tokyo and were performed in accordance with the guidelines for animal experiments of the Ministry of Education, Culture, Sports, Science and Technology, Japan.

### Perampanel treatment

Perampanel powder, which was provided by Eisai Co., Ltd., was suspended in a 0.5% W/V methyl cellulose (400 cP, WAKO) solution, and a volume of 4 μl per g of body weight was administered daily to the mice via oral gavage (p. o.). For the 14-day administration, 17-week-old mice received either 3.3, 6.6, 13.2 or 20 mg/kg (*n* = 5, 8, 5, and 6 AR2H mice, respectively) or 13.2 mg/kg (*n* = 5, AR2 mice) perampanel. For the 90-day administration, perampanel was administered to AR2 mice (26–28 weeks of age) at a dosage of 13.2 mg/kg/day for the first four days and then at 20 mg/kg/day for the remaining 86 days (*n* = 8). The control mice, AR2H (*n* = 7) and AR2 (*n* = 5) for the 14-day administration and AR2 (*n* = 7) for the 90-day administration, received oral gavage of the same volume of a 0.5% methyl cellulose solution for the same period.

### Performance analyses

#### Rotarod task

The mice were placed on a rotarod (Muromachi Kikai Co. LTD MK-610A), the speed of which linearly accelerated from four rpm to 40 rpm over 240 seconds. The maximum latency to fall from the rod out of three runs was recorded. The mice used in this study were well trained on the rotarod task prior to drug administration. *Grip strength.* The mice were held by their tails by the researcher and allowed to grasp the steel grip of the baseplate of a Grip Strength Meter (GSM) with their forepaws. The mice were then gently pulled backward until they released the steel grip. The average power (N) at the time of grip release of three trials was recorded. All behavioral measurements were conducted weekly by a researcher who was blinded to the drug administration information.

### Antibodies

The primary antibody included rabbit anti-TDP-43 (10782-2AP, ProteinTech Group, Inc.,), while the secondary antibodies included Alexa Fluor 488 chicken anti-rabbit IgG (A21441, Invitrogen).

### Reagents

Perampanel was obtained from Eisai Co., Ltd., methyl cellulose (400 cP) was purchased from WAKO, and TO-PRO-3 was purchased from lifetechnologies.

### Immunohistochemistry

Three hours after the last oral gavage administration, the brains and spinal cords of the mice were extracted under deep isoflurane anesthesia, incubated with 3.5% paraformaldehyde and 0.5% glutaraldehyde in phosphate-buffered saline (PBS) at 4 °C overnight and then immersed in a graded series of sucrose-PBS solutions (from 10% to 30% final sucrose concentration) at 4 °C. Ten-μm-thick serial sections were cut with a cryostat (Model LEICA CM1850; Leica), blocked with 10% skim milk in PBS, and incubated overnight at 4 °C with the rabbit anti-TDP-43 (ProteinTech Group, Inc., 1:200) primary antibody. The sections were then incubated with the Alexa Fluor 488 chicken anti-rabbit IgG (Invitrogen, 1:200 in PBS) secondary antibody for one hour at room temperature. Cell staining was performed with 0.5 μM TO-PRO-3. Immunostaining images were captured using a BIOREVO BZ-9000 fluorescence microscope (KEYENCE Co., Ltd.), and the number of cells with a diameter of ≥20 μm in the anterior horns (AHCs) was quantified in three sections per animal using ImageJ software (NIH). The immunoreactive signal intensity was analyzed with ImageJ software, with a grayscale background intensity of less than 20 (11.6 ± 0.55; mean ± s.e.m., max 19.9 gray). TDP-43 positivity was defined as an intensity threefold greater (≥60 gray) than that of the background intensity. The TDP-43 distribution pattern was classified as predominantly nuclear (N), cytoplasmic (C) or nucleo-cytoplasmic (N/C).

### Statistical analyses

Average data are presented as the mean and s.e.m. Statistical analyses were conducted using JMP 9 software (SAS Institute, Inc.). For statistical comparisons of two groups, we used the Wilcoxon rank sum test. Multi-group data were analyzed using one-way ANOVA followed by Tukey *post hoc* tests. Differences were considered significant when *p* < 0.05.

## Additional Information

**How to cite this article**: Akamatsu, M. *et al*. The AMPA receptor antagonist perampanel robustly rescues amyotrophic lateral sclerosis (ALS) pathology in sporadic ALS model mice. *Sci. Rep.* **6**, 28649; doi: 10.1038/srep28649 (2016).

## Supplementary Material

Supplementary Information
