# Supplementary material for: The AMPA receptor antagonist perampanel robustly rescues amyotrophic lateral sclerosis (ALS) pathology in sporadic ALS model mice
Source: Sci Rep. 2016 Jun 28;6:28649. doi: 10.1038/srep28649 (PMC4923865; doi:10.1038/srep28649)
Supplement: Supplementary Information [file srep28649-s1.pdf]

Title

The AMPA receptor antagonist perampanel robustly rescues amyotrophic lateral sclerosis (ALS) pathology in sporadic ALS model mice

Megumi Akamatsu[1,†], Takenari Yamashita[1,†], Naoki Hirose[1], Sayaka Teramoto[1], Shin Kwak[1,2,*]

[1] Center for Disease Biology and Integrative Medicine, Graduate School of Medicine, University of Tokyo, Bunkyo-ku, Tokyo, Japan

[2] Clinical Research Center for Medicine, International University of Health and Welfare, Ichikawa, Chiba, Japan

[†] These authors contributed equally to this work.

*Correspondence and requests should be addressed to S. K. (kwak-tky@umin.ac.jp).

Supplementary Figure 1

Figure S1

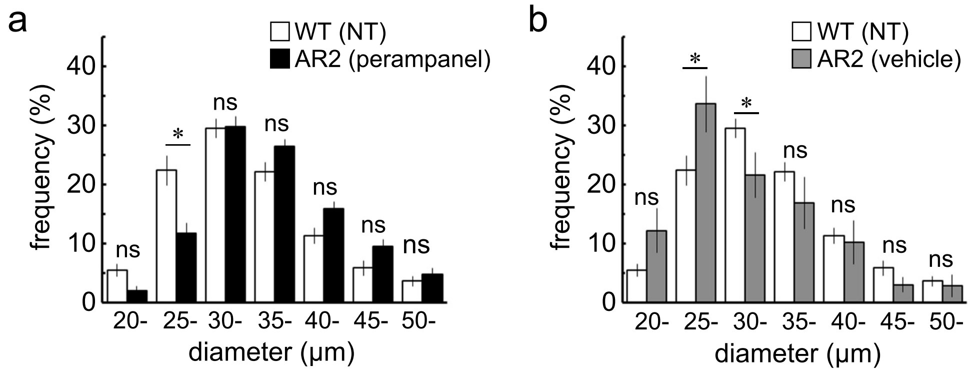

Supplementary Figure 1. Effects of 90-day administration of perampanel and vehicle on AHC size in AR2 mice. Frequency histogram of AHC diameter for perampanel-treated (**a**) and methyl cellulose-treated (**b**) 38-week-old AR2 mice compared to age-matched non-treated (NT) WT mice. The vertical axis indicates the proportion of the total number of AHCs with a diameter within each range. (**a**) The frequency peak was in the range of 30-35 µm in both the perampanel-treated AR2 and the WT mice. (**b**) The frequency peak for the vehicle-treated AR2 mice was in a smaller range (25-30 µm) than that for the WT mice (30-35 µm). All error bars represent the s.e.m. *$p < 0.05$, Wilcoxon rank sum test against WT (NT) for each diameter. The perampanel group included 8 mice, the vehicle group included 7, and the WT group included 5. AHCs were counted in three sections per mouse.
